# Supplementary material for: An open-source framework for large-scale, flexible evaluation of biomedical text mining systems
Source: J Biomed Discov Collab. 2008 Jan 29;3:1. doi: 10.1186/1747-5333-3-1 (PMC2276192; doi:10.1186/1747-5333-3-1)
Supplement: Additional file 1 — Evaluation platform source code. The additional file contains the software evaluation framework discussed in this paper. The most current release can be downloaded from SourceForge.net [1]. See the accompanying README file for instructions on installation and use. Also included in the distribution is a collection of UIMA wrappers for some commonly used BioNLP tools and annotated corpora. [file 1747-5333-3-1-S1.zip › bionlp_uima_repository-1.0.1/docs/api/edu/uchsc/ccp/uima/util/opendmap/AnnotationMap.html]

AnnotationMap (Center for Computational Pharmacology Util/UIMA API)


|  |  |  |
| --- | --- | --- |
| **Overview** \| **Package** \| **Class** \| **Tree** \| **Deprecated** \| **Index** \| **Help** | |  |
| PREV CLASS   **NEXT CLASS** | **FRAMES**    **NO FRAMES**     **All Classes** |  |
| SUMMARY: NESTED \| FIELD \| CONSTR \| METHOD | DETAIL: FIELD \| CONSTR \| METHOD |  |


---


## edu.uchsc.ccp.uima.util.opendmap Class AnnotationMap

```
java.lang.Object
  edu.uchsc.ccp.uima.util.opendmap.AnnotationMap
```

---

``` public class AnnotationMap extends Object ```

This class loads a translation map for use in moving from
CAS annotations to OpenDMAP input tokens and from OpenDMAP
output references to CAS annotations.

**Author:**
:   R. James Firby

---

**Constructor Summary**

`AnnotationMap()`
  
          Create an empty annotation map.

`AnnotationMap(String filename)`
  
          Create an annotation map from an XML file.


| **Method Summary** | |
| --- | --- |
| `String` | `getInputMentionReferenceType(String name)`             Get the type of DMAP Protege Frame ti use for the input token corresponding to this type of CAS annotation mention. |
| `String` | `getInputMentionSlotReferenceType(String mention, String slot)`             Get the slot name to use in the DMAP reference built to represent this CAS annotation mention slot. |
| `String` | `getOutputReferenceMentionType(edu.uchsc.ccp.opendmap.Reference reference)`             Get the type of CAS annotation mention that should be created to represent this DMAP reference. |
| `String` | `getOutputReferenceMentionType(String name)`             Get the type of CAS annotation mention that should be created to represent this DMAP reference type. |
| `edu.uchsc.ccp.opendmap.Reference` | `getOutputReferenceRoot(edu.uchsc.ccp.opendmap.Reference reference)`             Get the slot filler designated as the root for this reference. |
| `String` | `getOutputReferenceRootSlot(String cls)`             Get the name of the slot holding the root reference for references of this DMAP type. |
| `String` | `getOutputReferenceSlotMentionType(edu.uchsc.ccp.opendmap.Reference reference, edu.uchsc.ccp.opendmap.DMAPItem slot)`             Get the name to use for this reference slot in the CAS annotation mention being written back to the CAS. |
| `Vector<String>` | `getReferencesOfInterest()`             Get all of the types of the references of interest. |
| `boolean` | `hasInputMentionsOfInterest()`             Check whether any CAS annotations other than TokenAnnotations are to be used as input tokens (typically named entities). |
| `boolean` | `hasOutputReferencesOfInterest()`             Check whether any DMAP output references are to be written back into the CAS. |
| `boolean` | `isInputMentionOfInterest(String name)`             Check whether a specific CAS annotation mention is to become an input token. |
| `boolean` | `isInputMentionSlotIncluded(String mention, String slot)`             Check whether a particular slot of a CAS annotation mention should be included in the DMAP Protege Frame used for the input token. |
| `boolean` | `isOutputReferenceOfInterest(edu.uchsc.ccp.opendmap.Reference reference)`             Check whether a specific DMAP output reference should be written back into the CAS. |
| `boolean` | `isOutputReferenceOfInterest(String name)`             Check whether a specific DMAP output reference type should be written back into the CAS. |
| `boolean` | `isOutputReferenceSlotExcluded(String cls, String slot)`             Check whether an output reference slot should be included in the CAS annotation mention being written back to the CAS. |
| `boolean` | `isOutputReferenceSlotOfInterest(edu.uchsc.ccp.opendmap.Reference reference, edu.uchsc.ccp.opendmap.DMAPItem slot)`             Check whether this reference slot should be included in the annotation mention written back to the CAS. |
| `void` | `print(PrintStream stream, int indent)`             Print out the contents of this annotation map in a human-readable format. |
| `void` | `setInputMentionReferenceType(String name, String dmapType)`             Set the type of DMAP Protege Frame to use for the input token corresponding to this type of CAS annotation mention. |
| `void` | `setInputMentionSlotReferenceType(String mention, String slot, String dmapSlot)`             Set whether or not a CAS annotation mention slot should be included in DMAP input token references. |
| `void` | `setOutputReferenceMentionType(String dmapType, String mention)`             Set the type of CAS annotation mention that should be created to represent this DMAP reference type. |
| `void` | `setOutputReferenceRootSlot(String cls, String root)`             Set the name of the slot holding the root reference for references of this DMAP type. |
| `void` | `setOutputReferenceSlotExcluded(String cls, String slot, boolean excluded)`             Set whether an output reference slot should be included in the CAS annotation mention being written back to the CAS. |

**Methods inherited from class java.lang.Object**

`clone, equals, finalize, getClass, hashCode, notify, notifyAll, toString, wait, wait, wait`

**Constructor Detail**

### AnnotationMap

```
public AnnotationMap()
```

:   Create an empty annotation map. Mappings will need to be added explicitly.

---


### AnnotationMap

```
public AnnotationMap(String filename)
              throws ParserConfigurationException,
                     SAXException,
                     IOException
```

:   Create an annotation map from an XML file.

    **Parameters:**: `filename` - The name of the file holding the configuration. **Throws:**: `ParserConfigurationException` - If there is no good XML parser available: `SAXException` - If there is an XML syntax problem in the configuration file: `IOException` - If there is a problem reading the file


**Method Detail**

### hasInputMentionsOfInterest

```
public boolean hasInputMentionsOfInterest()
```

:   Check whether any CAS annotations other than TokenAnnotations are to be
    used as input tokens (typically named entities).

    :   **Returns:**: 'True' if there is at least one CAS annotation type to use

---


### isInputMentionOfInterest

```
public boolean isInputMentionOfInterest(String name)
```

:   Check whether a specific CAS annotation mention is to become an input
    token.

    :   **Parameters:**: `name` - The name of the CAS annotation mention **Returns:**: 'True' if this type of annotation mention should become a token

---


### getInputMentionReferenceType

```
public String getInputMentionReferenceType(String name)
```

:   Get the type of DMAP Protege Frame ti use for the input token corresponding
    to this type of CAS annotation mention.

    :   **Parameters:**: `name` - The name of the CAS annotation mention **Returns:**: The DMAP Protege Frame type to represent this annotation

---


### setInputMentionReferenceType

```
public void setInputMentionReferenceType(String name,
                                         String dmapType)
```

:   Set the type of DMAP Protege Frame to use for the input token corresponding
    to this type of CAS annotation mention.

    :   **Parameters:**: `name` - The name of the CAS annotation mention: `dmapType` - The DMAP Protege Frame type to represent this annotation

---


### isInputMentionSlotIncluded

```
public boolean isInputMentionSlotIncluded(String mention,
                                          String slot)
```

:   Check whether a particular slot of a CAS annotation mention should be included
    in the DMAP Protege Frame used for the input token.

    By default, no slots are included.

    :   **Parameters:**: `mention` - The CAS annotation mention name: `slot` - The CAS annotation mention slot name **Returns:**: 'True' if this slot should be carried into the DMAP input token

---


### getInputMentionSlotReferenceType

```
public String getInputMentionSlotReferenceType(String mention,
                                               String slot)
```

:   Get the slot name to use in the DMAP reference built to represent this CAS
    annotation mention slot.

    :   **Parameters:**: `mention` - The CAS annotation mention name: `slot` - The CAS annotation mention slot name **Returns:**: The DMAP reference slot name to use in the input token for this CAS mention

---


### setInputMentionSlotReferenceType

```
public void setInputMentionSlotReferenceType(String mention,
                                             String slot,
                                             String dmapSlot)
```

:   Set whether or not a CAS annotation mention slot should be included in
    DMAP input token references.

    :   **Parameters:**: `mentionThe` - CAS annotation mention name.: `slot` - The CAS annotation mention slot name.: `dmapSlot` - The DMAP reference name for the corresponding slot. Or null if this slot should be excluded.

---


### hasOutputReferencesOfInterest

```
public boolean hasOutputReferencesOfInterest()
```

:   Check whether any DMAP output references are to be written back into the CAS.

    :   **Returns:**: 'True' if there is at least one output reference of interest.

---


### isOutputReferenceOfInterest

```
public boolean isOutputReferenceOfInterest(String name)
```

:   Check whether a specific DMAP output reference type should be written
    back into the CAS.

    :   **Parameters:**: `name` - The name of the DMAP Reference frame type **Returns:**: 'True' if this reference should be written back

---


### isOutputReferenceOfInterest

```
public boolean isOutputReferenceOfInterest(edu.uchsc.ccp.opendmap.Reference reference)
```

:   Check whether a specific DMAP output reference should be written
    back into the CAS.

    :   **Parameters:**: `reference` - The reference to check **Returns:**: 'True' if this reference should be written into the CAS

---


### getOutputReferenceMentionType

```
public String getOutputReferenceMentionType(edu.uchsc.ccp.opendmap.Reference reference)
```

:   Get the type of CAS annotation mention that should be created to
    represent this DMAP reference.

    :   **Parameters:**: `reference` - The reference to check **Returns:**: The type of CAS annotation mention to create

---


### isOutputReferenceSlotOfInterest

```
public boolean isOutputReferenceSlotOfInterest(edu.uchsc.ccp.opendmap.Reference reference,
                                               edu.uchsc.ccp.opendmap.DMAPItem slot)
```

:   Check whether this reference slot should be included in the
    annotation mention written back to the CAS.

    By default, all slots are included (the opposite of input
    annotation to reference mappings).

    :   **Parameters:**: `reference` - The reference to be written back: `slot` - The name of the reference slot **Returns:**: 'True' if this slot should be included in the CAS mention

---


### getOutputReferenceSlotMentionType

```
public String getOutputReferenceSlotMentionType(edu.uchsc.ccp.opendmap.Reference reference,
                                                edu.uchsc.ccp.opendmap.DMAPItem slot)
```

:   Get the name to use for this reference slot in the CAS annotation mention
    being written back to the CAS.

    :   **Parameters:**: `reference` - The reference being written back: `slot` - The name of the reference slot **Returns:**: The name to use for the corresponding CAS slot

---


### getOutputReferenceRoot

```
public edu.uchsc.ccp.opendmap.Reference getOutputReferenceRoot(edu.uchsc.ccp.opendmap.Reference reference)
```

:   Get the slot filler designated as the root for this reference. If there
    is no designated slot, then just return the reference.

    The root of a reference is the slot value that anchors the reference in
    the parsed text and disambiguates the reference from other references of
    the same type that were found. For example, a slot that holds the "verb" for
    a reference might be a natural root.

    :   **Parameters:**: `reference` - The reference to find the root of. **Returns:**: The root for that reference

---


### getReferencesOfInterest

```
public Vector<String> getReferencesOfInterest()
```

:   Get all of the types of the references of interest.

    :   **Returns:**: The names of the reference types of interest.

---


### getOutputReferenceMentionType

```
public String getOutputReferenceMentionType(String name)
```

:   Get the type of CAS annotation mention that should be created to
    represent this DMAP reference type.

    :   **Parameters:**: `name` - The name of the DMAP reference type of interest. **Returns:**: The corresponding CAS annotation mention type to create.

---


### setOutputReferenceMentionType

```
public void setOutputReferenceMentionType(String dmapType,
                                          String mention)
```

:   Set the type of CAS annotation mention that should be created to
    represent this DMAP reference type.

    :   **Parameters:**: `dmapType` - The DMAP reference type of interest.: `mention` - The CAS annotation mention type to create for this DMAP type.

---


### getOutputReferenceRootSlot

```
public String getOutputReferenceRootSlot(String cls)
```

:   Get the name of the slot holding the root reference for references of
    this DMAP type.

    :   **Parameters:**: `cls` - The type of the DMAP reference. **Returns:**: The name of the DMAP reference slot holding the root for this type of reference.

---


### setOutputReferenceRootSlot

```
public void setOutputReferenceRootSlot(String cls,
                                       String root)
```

:   Set the name of the slot holding the root reference for references of
    this DMAP type.

    :   **Parameters:**: `cls` - The type of the DMAP reference.: `root` - The name of the DMAP reference slot holding the root for this type of reference.

---


### isOutputReferenceSlotExcluded

```
public boolean isOutputReferenceSlotExcluded(String cls,
                                             String slot)
```

:   Check whether an output reference slot should be included in the CAS
    annotation mention being written back to the CAS.

    :   **Parameters:**: `cls` - The DMAP reference type: `slot` - The DMAP reference slot name **Returns:**: 'True' if this slot should be excluded from the CAS

---


### setOutputReferenceSlotExcluded

```
public void setOutputReferenceSlotExcluded(String cls,
                                           String slot,
                                           boolean excluded)
```

:   Set whether an output reference slot should be included in the CAS
    annotation mention being written back to the CAS.

    :   **Parameters:**: `cls` - The DMAP reference type: `slot` - The DMAP reference slot name: `excluded` - 'True' if this slot should be excluded from the CAS

---


### print

```
public void print(PrintStream stream,
                  int indent)
```

:   Print out the contents of this annotation map in a human-readable format.

    :   **Parameters:**: `stream` - The stream to print to.: `indent` - An indent amount.


---


|  |  |  |
| --- | --- | --- |
| **Overview** \| **Package** \| **Class** \| **Tree** \| **Deprecated** \| **Index** \| **Help** | |  |
| PREV CLASS   **NEXT CLASS** | **FRAMES**    **NO FRAMES**     **All Classes** |  |
| SUMMARY: NESTED \| FIELD \| CONSTR \| METHOD | DETAIL: FIELD \| CONSTR \| METHOD |  |


---
